# 肺癌胸腔镜术后并发症的防治策略

**DOI:** 10.3779/j.issn.1009-3419.2018.03.24

**Published:** 2018-03-20

**Authors:** 友生 毛, 捷 赫

**Affiliations:** 国家癌症中心/中国医学科学院北京协和医学院肿瘤医院胸外科 Cancer Hospital of the Chinese Academy of Medical Sciences

肺癌目前已经成为国内发病率和死亡率最高的恶性肿瘤^[1]^，严重威胁着人们的健康。外科手术作为肺癌综合治疗中最主要的一环，近年来发展迅速，尤其是胸腔镜技术的应用和普及起到了重要的作用。胸腔镜手术与传统的开胸手术相比，有着创伤少、疼痛轻、术后康复快等优势，但术后并发症的发生，无疑会延长患者住院时间并抵消胸腔镜的优势。如何有效地降低肺癌患者胸腔镜术后并发症的发生率，更好更快地促进患者的康复，成为当下医护人员关注的焦点之一。受本刊报道的“胸腔镜肺癌切除术后患者住院时间（ > 7天）延长的原因分析”的启示，本文就胸腔镜术后并发症的防治进行述评。

为了对术后并发症进行准确评估，Clavien等^[2]^在1992年建立了一套并发症的分级系统，并在2004年由Dindo等^[3]^进行改进，将术后并发症依据严重程度以及是否需要针对并发症进行相应的药物或手术治疗，由轻到重依次分为Ⅰ级-Ⅴ级，Ⅰ级：无需药物、手术、内镜或放射治疗的术后出现的异常改变，但包括需要止吐药、解热药、止痛药、电解质和物理治疗的并发症，还包括需要在床旁行开放引流的伤口感染；Ⅱ级：需要除Ⅰ级所用药物以外的药物治疗，还包括输血和全胃肠外营养；Ⅲ级：需要行手术、内镜、放射治疗等干预措施的术后并发症，Ⅲa级：干预措施不需要在全麻下进行，Ⅲb级：干预措施需要在全麻下进行；Ⅳ级：危及生命的并发症，包括中枢神经系统并发症、需要重症监护或至重症监护病房处理的并发症，Ⅳa级：单个器官功能障碍（包括透析），Ⅳb级：多器官功能障碍；Ⅴ级：死亡。其中，术后的各级并发症中Ⅱ级并发症出现的频率最高，可占全部的63.9%^[4]^。文献报道，胸腔镜肺癌术后的患者出现并发症率波动于2%-40%^[5]^，而一项国内的研究报道胸腔镜术后并发症发生率为15%^[6]^。肺癌胸腔镜术后常见的Ⅰ级-Ⅱ级并发症主要包括：轻度肺部感染、肺不张、 > 5天的肺漏气、胸腔积液、心率失常等。Ⅲ级-Ⅳ级严重并发症包括术中或术后胸腔出血，严重肺部感染并发继发性呼吸衰竭，支气管胸膜瘘，脓胸，心脑血管并发症（心衰、心梗、脑出血、脑梗塞、肺动脉栓塞等）。术后并发症的产生会造成患者的住院时间延长，住院费用增多，抗生素使用时间延长及患者心理压力增大等一系列负面后果，严重者甚至导致患者死亡。

既往研究显示，术前低肺功能（FEV_1_ < 70%pred、低DLCOppo）的患者术后出现并发症的概率会显著升高^[7-9]^，另外，高龄、长期吸烟史及术前合并症较多的患者（高血压病、慢性阻塞性肺疾病、糖尿病、各种心脏病，心梗和脑梗病史等）术后出现并发症的几率明显增高^[10-12]^。因此，针对高危患者，如何有效地防治术后并发症的发生，对促进患者术后康复极为重要。其防治策略包括三个方面：术前要全面评估患者手术风险并作出预案；术中要精细操作并恰当处理可能出现并发症的问题，术后要规范监测并及时发现和处理出现的并发症，最大程度改善患者的预后，促进患者康复。

## 1 术前风险评估和准备

肺癌患者术前风险评估包括两个方面，一是术前肿瘤临床分期评估，另一个则是围手术期风险评估。肿瘤期别越晚，手术越大，创伤越大，手术风险越大；患者年龄越大，合并症越多，风险越大。因此，两者的评估都很重要，两者风险也会叠加。术前评估分为三个步骤，首先要详细询问病史和进行必要的体格检查，包括肿瘤方面的症状和既往各种病史，尤其是与心、肺、肝、脑、肾等重要器官相关的病史，所用药物及治疗史。从中得到可以用于风险评估的相关合并症和可能出现风险的信息。其次需要应完善所有的有关分期和风险评估的检查：包括胸腹部计算机断层扫描（computed tomography, CT）、全身全身正电子发射计算机断层显像（positron emission tomography/CT, PET/CT）、脑核磁、骨扫描、肝肾功能、凝血功能、心肺功能等系列检查，特殊检查包括气管镜、食管镜、运动心肺功能、超声心动、冠脉造影及核素肺通气和灌注扫描等。最后也是最重要的是判读所有从病史和检查中得来的信息，通过综合分析评估判断出患者围手术期风险大小并做出必要预案来防范风险。胸部高分辨增强CT是最必需的检查，它不仅可提供肿瘤原发灶的细节影像特征以做出诊断，也可了解纵隔淋巴结转移情况，尤其是纵隔淋巴结大小、有无钙化、与血管关系等，是判断VATS肺癌手术中风险的重要信息。心肺功能检查对于胸腔镜肺癌手术患者也非常重要，除了要研读心肺功能报告之外，更要判断报告指标的准确性，如果患者配合欠佳或完全不会配合会导致心肺功能检查指标不合格，通常需要做更客观的检查比如运动心肺功能检查或简单的爬楼试验。一般患者如果FEV_1_ > 2.0 L，MVV% > 50%，可以耐受一侧全肺切除术；FEV_1_ > 1.0 L，MVV% > 40%，可以耐受肺叶切除；FEV_1_ > 0.6 L，MVV% > 35%，可以耐受肺段与楔形切除^[13-15]^。一口气可登上3楼（约11 m）者可耐受肺叶切除术，可登上5楼（约18.4 m）者，可耐受全肺切除。患者登楼梯能力与术后死亡率之间明显呈负相关^[16-18]^。对于慢性阻塞性肺疾病或心肺功能异常的患者，术前除需要进行健康宣教外，吸烟者要尽早戒烟和进行呼吸功能锻炼，并在医护人员指导下学习术后深呼吸和咳痰技巧，必要时给予化痰、平喘、镇咳、雾化等药物治疗，如有肺部感染，宜选用敏感抗生素进行抗感染治疗。对于有高血压病、糖尿病、心脏病等合并症的患者，术前应请相关科室会诊和评估，制定用药方案，将血压，血糖等控制在可以耐受手术的范围，避免使用与手术相冲突的药物（如阿司匹林、氯吡格雷、利血平等）。此外，冠心病的患者术前应完善冠状动脉CT检查或冠脉造影，明确冠脉狭窄程度，如果冠脉狭窄超过70%以上，一般需要先放置支架或冠脉搭桥手术治疗。术前有心律失常的患者应行24 h动态心电图检查，如果心律失常超过5次/分钟以上，需要先用药物治疗后再手术。

## 2 术中精细操作

胸腔镜肺癌手术的术中操作分为麻醉和手术两个部分，麻醉是保证平稳完成一个精美手术操作过程不可缺少的重要环节。术中麻醉医师应根据患者的实际情况选用合适的气管插管，为保证麻醉插管到位并起到良好隔离效果，建议利用儿童支气管镜将气管插管调整并确认已置于合适的位置，以保证患者术中的良好通气和隔离作用；否则由于双腔插管的位置不合适会导致术侧肺不能塌陷或塌陷不完全，影响手术进程，甚至导致术中血管意外损伤出血和术后漏气的发生。另外，选用恰当的麻醉药物，控制术中补液量，保证患者在术中生命体征平稳以及血流动力学的稳定也非常重要。过量补液会导致术后肺水肿和呼吸衰竭发生率增加。

手术方面，胸外科医师术前应当充分仔细阅片，熟悉掌握手术操作区域的解剖结构，对可能存在的解剖变异提前标注，对术中预估到的难点做好充足的准备。术中应当选用合适的器械和手术入路，不能思维僵化和拘泥于开孔多少的限制，保证手术安全和根治才是最终目的。从本刊发表的北京大学附属肿瘤医院戴亮等报道胸腔镜肺癌切除术后患者住院时间（ > 7天）延长的原因分析中看出，胸腔镜术后漏气是延长住院时间的重要原因，占术后总并发症人数的22.6%。因此，术后肺持续漏气是肺叶切除术后最常见并发症之一，其发生率为3%-25%，常造成术后胸腔引流管留置时间延长而导致住院时间延长和住院费用增加^[19-21]^。因此，如何避免术中肺组织损伤以防止术后漏气非常重要。发育完全的肺裂可采用电钩处理，但对于先天发育欠佳的肺裂，建议尽量使用腔镜切割缝合器处理，对于慢性阻塞性肺疾病患者，建议加用耐维处理肺组织，避免术后漏气的发生。术中利用电钩和超声刀准确和精细地进行组织的游离时，要避免损伤周围重要结构如大的血管、气管、食管和神经，尽量保持术野清晰以减少术中出血和操作失误。对于血管、支气管的常规处理应选用厚度合适的钉仓，而对于细小的动静脉分支，必要时可行结扎处理，以避免残端渗血；手术结束前应当对胸腔进行冲洗，并在麻醉医师充分吸痰后恢复患侧通气，仔细观察胸腔内有无气泡逸出。对于胸膜表面的细小破损，可在术后肺叶充分复张后自行闭合，而对于肺叶表面较大的破损及支气管残端的漏气，建议用可吸收线加以缝合以缩短术后漏气时间。此外，还应恰当地放置引流管，使得术后能够充分引流，并在关胸前患侧充分通气，使得术侧剩余肺叶组织充分复张。

## 3 术后严密观察与早期处理

术后除常规进行生命体征监护外，还要密切关注患者各种引流及生命体征的变化。对术后出现心率失常、低氧血症的患者要早发现、早治疗。对于慢性阻塞性肺疾病和支气管成形术后的患者，术后雾化吸入，促进患者咳嗽排痰极为重要。对于全肺切除术后、高龄及既往心脏病史的患者要严格控制补液量，避免出现肺水肿、心衰等严重的并发症。对于顽固性低氧血症的患者，可由常规的鼻导管吸氧换为储氧面罩、无创BiPAP通气，必要时可行气管插管或气管切开呼吸机辅助呼吸。术后适度应用预防性抗生素，观察术后体温、血象及胸片的动态变化非常重要。通过每日查房的听诊和床旁胸片，关注患者双肺呼吸音和肺复张的变化，如有大面积的啰音或呼吸音减弱/消失，则需要高度警惕肺部感染、心衰或肺不张等并发症的发生。另外，要密切关注胸腔闭式引流情况，观察患者在深呼吸、咳嗽时胸瓶内有无气泡逸出，引流液的颜色有无变化。如出现乳糜样胸水或大量血性胸水应及时处理。对于体温 > 38.5 oC，血常规显示白细胞 > 10.0×10^9^/L，胸片见肺部渗出或实变影的患者，需考虑肺部感染可能。肺部感染的患者，应及时做痰培养，在明确病原菌后尽早足量足疗程使用敏感抗生素。而对于术后出现的肺不张，则需要支气管镜下吸痰，解除呼吸道的梗阻。乳糜胸的患者应禁食，行肠外营养补充治疗。对于经初步治疗后临床症状、体征能够保持稳定、无进一步恶化征象者；或乳糜胸出现相对较晚，或胸水的增长小于1, 000 mL/d，并呈减少趋势的患者，均可考虑保守治疗，大多数患者经保守治疗后可以痊愈。而对于保守治疗10天以上，胸水量仍难以控制，或每日引流量大于1, 000 mL的乳糜胸患者，则需要考虑胸腔镜或开胸探查结扎胸导管^[22, 23]^。对于出现大量血性胸水的患者，要高度警惕术后出血，需监测生命体征，复查血常规以明确术前及术后的血红蛋白水平的变化，对于出现心率加快、血压下降等早期休克征象或出现进行性血胸的患者，应积极开胸探查，以确保患者的安全。

总之，肺癌胸腔镜手术并发症的防治是一个系统工程，它牵涉到多个学科的配合，缺一不可。因此，胸外科，麻醉科、重症监护和护理各个环节的密切配合极为重要。术前详细的风险评估和准备，术中仔细操作和术后的严密观察与处理是防治胸腔镜肺癌术后并发症发生的重要策略。
